# Obesity: Pathophysiology and Intervention

**DOI:** 10.3390/nu6115153

**Published:** 2014-11-18

**Authors:** Yi Zhang, Ju Liu, Jianliang Yao, Gang Ji, Long Qian, Jing Wang, Guansheng Zhang, Jie Tian, Yongzhan Nie, Yi Edi. Zhang, Mark S. Gold, Yijun Liu

**Affiliations:** 1 School of Life Science and Technology, Xidian University, Xi’an, Shaanxi 710071, China; E-Mails: liuju@life.xidian.edu.cn (J.L.); yaojianliang@life.xidian.edu.cn (J.Y.); wangjingwroolafd@163.com (J.W.); zhangguansheng66@126.com (G.Z.); tian@ieee.org (J.T.); 2 Department of Psychiatry & McKnight Brain Institute, University of Florida, 1149 South Newell Drive, Gainesville, FL 32610, USA; E-Mails: yizhang@ufl.edu (Y.E.Z.); msgold@ufl.edu (M.S.G.); 3 Xijing Gastrointestinal Hospital, the Fourth Military Medical University, Xi’an, Shaanxi 710032, China; E-Mails: jigang@fmmu.edu.cn (G.J.); nieyongzhan@gmail.com (Y.N.); 4 Department of Biomedical Engineering, Peking University, Beijing 100871, China; E-Mail: qianlong2008@yeah.net; 5 Malcom Randall Veterans Affairs Medical Center, Gainesville, FL 32608, USA; 6 Department of Psychology, Southwest University, Chongqing 400715, China

**Keywords:** obesity, food addiction, neuroendocrinology, neuroimaging, reward-saliency, motivation-drive, learning/memory circuit, inhibitory control-emotional regulation-executive control, bariatric surgery, fecal microbiota transplantation

## Abstract

Obesity presents a major health hazard of the 21st century. It promotes co-morbid diseases such as heart disease, type 2 diabetes, obstructive sleep apnea, certain types of cancer, and osteoarthritis. Excessive energy intake, physical inactivity, and genetic susceptibility are main causal factors for obesity, while gene mutations, endocrine disorders, medication, or psychiatric illnesses may be underlying causes in some cases. The development and maintenance of obesity may involve central pathophysiological mechanisms such as impaired brain circuit regulation and neuroendocrine hormone dysfunction. Dieting and physical exercise offer the mainstays of obesity treatment, and anti-obesity drugs may be taken in conjunction to reduce appetite or fat absorption. Bariatric surgeries may be performed in overtly obese patients to lessen stomach volume and nutrient absorption, and induce faster satiety. This review provides a summary of literature on the pathophysiological studies of obesity and discusses relevant therapeutic strategies for managing obesity.

## 1. Introduction

Obesity is a serious global epidemic and poses a significant health threat to humans. The prevalence of obesity is increasing not only in adults, but also among children and adolescents [1]. Obesity is associated with increased risks for atherosclerotic cerebrovascular disease, coronary heart disease, colorectal cancer, hyperlipidemia, hypertension, gallbladder disease, and diabetes mellitus, as well as a higher mortality rate [2]. It places a remarkable burden on societal health expenditure [3]. Causes of obesity are multitude, and the etiology is not well known. Obesity is at least in part attributable to overconsumption of calorie-dense foods and physical inactivity [1,2,4]. Other factors such as personality traits, depression, side effects of pharmaceuticals, food addiction, or genetic predisposition may also contribute.

This article provides a broad overview of the literature on obesity from multiple perspectives, including epidemiological investigation, food addiction, endocrine, and neuroimaging studies on brain circuits associated with eating and obesity. It presents the currently debatable notion of food addiction in obesity and hopes to generate more discussion and research efforts to validate this idea. The review also offers a detailed update on many of the most recent neuroimaging investigations on certain critical neural circuits implicated in appetite and addiction control. This update will help readers gain a better understanding of the CNS regulation of eating behavior and obesity, and the overlapping neuropathophysiological bases for addiction and obesity. Last but not the least, the end section of the paper summarizes the relevant therapeutic approaches for managing obesity and introduces exciting new treatment strategies.

## 2. Epidemiological Studies

The prevalence of obesity has skyrocketed in most western countries over the past 30 years [5]. The United States and the United Kingdom have seen large increases since the 1980s, while many other European countries reported smaller increases [3]. The WHO estimated that approximately 1.5 billion adults over the age of 20 years old were overweight worldwide, and 200 million males and 300 million females were obese in 2008 [6]. The WHO also projects that approximately 2.3 billion adults will be overweight and more than 700 million obese by the year 2015 [6]. The statistics in children show an alarming upward trend. In 2003, 17.1% of children and adolescents were overweight, and 32.2% of adults were obese in the United States alone [2,7]. It is estimated that 86.3% of Americans may be overweight or obese by 2030 [8]. Globally, nearly 43 million children under the age of five years were overweight in 2010 [9]. The obesity phenomenon is also drawing attention in developing countries [6]. The Chinese government disclosed that the total obese population was over 90 million and the overweight more than 200 million in 2008. This number could rise to more than 200 million obese and 650 million overweight in the next 10 years [3].

Obesity causes and worsens co-morbid illnesses, decreases quality of life, and increases risk of death. For instance, over 111,000 deaths each year in the United States are obesity-related [10]. Epidemiological studies indicate that obesity contributes to the higher incidence of and/or death from cancers of the colon, breast (in postmenopausal women), endometrium, kidney (renal cell), esophagus (adenocarcinoma), gastric cardia, pancreas, gallbladder, and liver, and possibly other types. Approximately 15%–20% of all cancer deaths in US are linked to overweight and obesity [11]. Adams *et al*. [12] investigated the risk of death in a prospective cohort of more than 500,000 US males and females with a 10 year follow-up. Among patients who had never smoked, the risk of death was found to increase by 20%–40% in the overweight and by two- to three-fold in the obese compared with the normal weight subjects [12].

Among numerous factors influencing obesity, overconsumption of caloric dense foods is one major culprit. Currently, in developed countries and developing countries alike, the food industry is rather successful in the mass production and marketing of calorie-dense foods [13]. Such foods are made readily available in grocery stores, shops, schools, restaurants, and homes [14]. There was a 42% per capita increase in the consumption of added fats and a 162% increase for cheese in the United States from 1970 to 2000. In contrast, consumption of fruits and vegetables only increased by 20% [15]. High-calorie foods present motivational and reward cues that likely trigger overconsumption [16]. Brain imaging studies demonstrate hyperactivation in the gustatory cortex (insula/frontal operculum) and oral somatosensory regions (parietal and rolandic operculum) in the obese relative to normal weight subjects in response to anticipated intake and consumption of palatable foods, and hypoactivation in the dorsal striatum and reduced striatal D2 dopamine receptor density in response to consumption of palatable foods [17]. These findings [17] indicated a relationship between abnormalities in food reward and an increased risk for future weight gain, suggesting greater weight gain for participants in an unhealthy food environment [4].

## 3. Binge Eating and Food Addiction

### 3.1. Binge Eating

Disordered eating and unhealthy weight control practices are widespread among adolescents, which may place them at risk for an eating disorder. Eating disorders are associated with a chronic course, high recidivism rates, and numerous medical and psychological comorbidities. Therefore, the need for early identification and prevention of eating disorders becomes an important issue that requires more attention from primary care services [18,19].

Binge-eating disorder (BED) is the most common eating disorder in adults. The disorder affects an individual’s emotional and physical health and is an important public health problem [20,21]. About 2.0% of men and 3.5% of women bear this illness lifelong—statistics higher than for the commonly recognized eating disorders anorexia nervosa and bulimia nervosa [20]. BED is characterized by binge eating without subsequent purging episodes and an association with the development of severe obesity [22]. People who are obese and have BED often became overweight at an earlier age than those without the disorder [23]. They might also lose and gain back weight more often, or be hypervigilant about gaining weight [23]. Binging episodes usually include foods that are high in fat, sugar, and/or salt, but low in vitamins and minerals, and poor nutrition is common in people with BED [21,23]. Individuals are often upset about their binge eating and may become depressed. Obese individuals with BED are at risk for common comorbidities associated with obesity such as type 2 diabetes mellitus, cardiovascular disease (*i.e.*, high blood pressure and heart disease), gastrointestinal issues (e.g., gallbladder disease), high cholesterol levels, musculoskeletal problems, and obstructive sleep apnea [20,21]. They often have a lower overall quality of life and commonly experience social difficulties [21]. Most people with binge eating disorder have tried to control it on their own, but fail at the attempt for an extended period of time.

### 3.2. Food Addiction

BED exhibits characteristics typically seen with addictive behaviors (e.g., diminished control and continued use of substances despite negative consequences). Evidence is accumulating in support of addiction conceptualizations of problematic eating [24]. Animal models suggest a relationship between binge eating and addiction-like food consumption. Rats given food rich in highly palatable or processed ingredients (e.g., sugar and fat) display behavioral indicators of binge eating, such as consuming elevated quantities of food in short time periods and seeking out highly processed foods regardless of negative consequences (*i.e*., electric foot shocks) [25,26]. Beyond behavioral alterations, the rats also demonstrate neural changes implicated in drug addiction, such as reduced dopamine D2 receptor availability [26]. These data suggest that BED may be one manifestation of food addiction [24].

Whether or not obesity involves food addiction in some obese people is still debatable. Growing data favor the idea that excess food intake may drive addictive behaviors [27]. Certain addictive behaviors, such as failed attempts to reduce food intake or continued feeding in spite of negative fallout, manifest in troubled eating patterns [27]. The brain also appears to respond to highly palatable foods in some similar fashions as it does to addictive drugs [28]. The current hypothesis is that certain foods or ingredients added to foods might trigger the addictive process in susceptible people [29]. The addictive process is more or less viewed as a chronic relapsing issue dependent upon factors that elevate cravings for food or food-related substances and heighten the state of pleasure, emotion, and motivation [30,31,32,33,34].

The Yale Rudd Center for Food Policy and Obesity, a non-profit research and public policy organization, reported in 2007 striking similarities in the use and withdrawal patterns of sugar and classic drugs of abuse, as well as reciprocal correlations between food intake and substance abuse (e.g., people tend to gain weight when they stop smoking or drinking). This raises the possibility that palatable foods and classic addictive substances may compete for similar neurophysiological pathways [35,36]. The Rudd Center helped create the Yale Food Addiction Scale (YFAS), which is designed to identify signs of addiction exhibited towards certain types of food with high fat and sugar contents [37,38]. Gearhardt and her colleague [39] have recently examined brain activation to food cues in patients with various scores on the food addiction scale. The patients were either signaled for impending delivery of a chocolate milkshake or a tasteless control solution, or were given a chocolate milkshake or a tasteless solution [39]. The results showed an association between higher food addiction scores and increased activation of brain regions encoding motivation in response to food cues, such as the amygdala (AMY), anterior cingulate cortex (ACC), and orbitofrontal cortex (OFC). It was concluded that addictive individuals are more likely to react to substance cues, and that the anticipation of a reward when a cue is noticed could contribute to compulsive eating [39]. In general, food addiction is not well defined and may be associated with substance use disorders [40] and eating disorders. It is noteworthy that the DSM-5 has proposed revisions recognizing binge eating disorder [41] as a free-standing diagnosis and renaming the category of Eating Disorders as Eating and Feeding Disorders.

### 3.3. Prader-Willi Syndrome (PWS)

Prader-Willi syndrome (PWS) is a genetic imprinting disorder that results in profound hyperphagia and early childhood onset obesity [42]. PWS patients display many addictive eating behaviors [43]. Neuroimaging studies in this naturally occurring human eating disorder model may uncover neurophysiological mechanisms governing food addiction or loss of control of eating in general. One characteristic of the disease is a marked obsessive drive to overeat not only food but also neutral non-food objects. Excessive and pathologic reinforcement produced by the ingested items themselves might contribute to this phenomenon [42,43,44,45,46,47,48,49,50]. Functional neuroimaging studies have investigated the abnormalities of eating-related neural circuitry using visual cues in PWS patients [44]. In response to visual high- *versus* low-calorie food stimulation after glucose administration, the PWS patients exhibited a delayed signal reduction in the hypothalamus (HPAL), insula, ventromedial prefrontal cortex (VMPFC), and nucleus accumbens (NAc) [44], but hyperactivity in limbic and paralimbic regions such as the AMY that drive eating behavior and in regions such as the medial prefrontal cortex (MPFC) that suppress food intake [47,51]. Increased activation in the HPAL, OFC [46,51,52], VMPFC [49], bilateral middle frontal, right inferior frontal, left superior frontal, and bilateral ACC regions was also observed [48,52,53]. Our group performed a resting-state fMRI (RS-fMRI) study combined with functional connectivity (FC) analysis and identified the alterations of FC strength among the brain regions in the default mode network, core network, motor sensory network, and prefrontal cortex network, respectively [53]. We recently utilized RS-fMRI and Granger causality analysis techniques to investigate the interactive causal influences among key neural pathways underlying overeating in PWS. Our data revealed significantly enhanced causal influences from the AMY to the HPAL and from both the MPFC and ACC to the AMY. In summary, PWS is the extreme end of human cases of obesity and uncontrollable eating behaviors. Investigation of the neurophysiological underpinning of PWS and its association with substance dependence may aid better understanding of appetite control and food addiction [39,43].

## 4. Hormones and Gut Peptides

Many peripheral hormones participate in central nervous system (CNS) control of appetite and food intake, food reward, or addiction. Both palatable foods and drugs are able to activate the mesolimbic dopamine (DA) reward system essential for addiction regulation in humans and animals [43,54,55,56,57,58]. Hunger and satiety signals from adipose tissue (leptin), the pancreas (insulin), and the gastrointestinal tract (cholecystokinin (CCK), glucagon-like peptide-l (GLP-1), peptide YY3-36 (PYY3-36), and ghrelin) are involved in relaying information about energy status through the neural hormonal gut-brain axis primarily targeting the hypothalamus (HPAL) and brainstem [58], and may directly or indirectly interact with the midbrain DA pathways to impact feeding [59,60,61].

### 4.1. Leptin

An anorexigenic hormone synthesized from adipose tissue, leptin regulates lipid metabolism by stimulating lipolysis and inhibiting lipogenesis [62]. Leptin crosses the blood-brain barrier via a saturable transport system and communicates the periphery metabolic status (energy storage) to the hypothalamic regulatory centers [63]. Once bound to its central receptor, leptin down-regulates appetite-stimulating neuropeptides (e.g., NPY, AgRP) while up-regulating anorexigenic alpha-melanocyte-stimulating hormone, cocaine- and amphetamine-regulated transcript, and corticotropin-releasing hormone [63]. Genetic defects in leptin and leptin receptors result in severe early onset obesity in children [64]. Leptin concentration in the blood is elevated in obesity, promoting a leptin resistance that renders the elevated leptin futile in curbing appetite and obesity. The presence of leptin resistance may offer a partial explanation for severe hyperphagia in PWS patients whose serum leptin levels are quite high [64]. People in the process of becoming addicted to food may also have leptin resistance, which could lead to overeating [65]. Leptin influence on addictive and non-addictive eating behaviors may be partially mediated through the regulation of the mesolimbic and/or nigrostriatal DA pathways. As one fMRI study demonstrated, supplemented leptin diminished food reward and enhanced satiety during food consumption by modulating neuronal activity in the striatum in leptin-deficient human subjects [66]. Leptin monotherapy, however, has not been successful in reducing food intake and weight gain in obese humans as originally hoped, possibly due to preexisting leptin resistance in obesity [67]. On the other hand, a low-dose leptin supplement may be useful for tempering the reward value of food [68] and helping to maintain lost weight.

### 4.2. Insulin

Insulin is a pancreatic hormone critical for maintenance of glucose homeostasis. Insulin levels rise after a meal to keep blood glucose in check. The excess glucose is converted and stored in the liver and muscle as glycogen, and as fat in adipose tissues. Insulin concentrations vary with adiposity, and the amount of visceral fat is negatively correlated with insulin sensitivity [69]. Fasting and postprandial insulin are higher in obese than in lean individuals [70]. Insulin can penetrate the blood-brain barrier and binds to receptors in the arcuate nucleus of the hypothalamus to decrease food intake [71]. Central insulin resistance may occur in obesity, similarly to the central leptin resistance that is thought to be consequential to high fat consumption or obesity development [72,73]. A positron emission tomography (PET) study identified insulin resistance in the striatum and insula areas of the brain and suggested that such a resistance may require higher brain insulin levels in order to adequately experience the reward and the interoceptive sensations of eating [74]. Like leptin, insulin is capable of modulating the DA pathway and associated eating behaviors. Leptin and insulin resistance in the brain DA pathways may result in heightened intake of palatable foods as compared to leptin- and insulin-sensitive conditions in order to generate a sufficient reward response [75].

The interplay between the central and peripheral hormonal signaling pathways is complex. For example, ghrelin stimulates dopaminergic reward pathways, while leptin and insulin inhibit these circuits. Moreover, signaling circuits in both the HPLA and the ARC receive afferent peripheral sensory signals and project and relay the information to other regions of the brain, including the midbrain dopaminergic reward center [31].

### 4.3. Ghrelin

Mainly secreted by the stomach, ghrelin is an orexigenic peptide that acts on hypothalamic neurons containing ghrelin receptors to exert central metabolic effects [76]. Ghrelin increases food intake in humans by both peripheral and central mechanisms involving interplay between the stomach, the HPAL, and the hypophysis [77,78]. Ghrelin appears to be an initiator of feeding with peak serum levels prior to food ingestion and reduced levels thereafter [79]. Ghrelin may chronically impact energy equilibrium, considering that prolonged ghrelin administration enlarges adiposity [77,80]. Serum ghrelin levels are lower in the obese relative to normal weight individuals and characteristically increase with obesity reduction, demonstrating a negative correlation with high BMIs [81,82]. Ghrelin activates the brain regions important for hedonic and incentive responses to food cues [83]. This includes activation of dopamine neurons in the VTA and increased dopamine turnover in the NAc of the ventral striatum [84]. The effects on reward processing in the mesolimbic dopaminergic pathway may be an integral part of ghrelin’s orexigenic action [83], supported by evidence that blocking ghrelin receptors in the VTA decreases food intake [84].

### 4.4. Peptide YY (PYY)

PYY is a short, 36-amino acid peptide made in the ileum and colon in response to feeding. Following food ingestion, PYY is released from the L-cells in the distal segment of the small gut. It reduces the rate of intestinal motility and gallbladder and gastric emptying and therefore decreases appetite and augments satiety [85,86]. PYY acts via the vagal afferent nerves, the NTS in the brainstem, and the anorexinergic cycle in the hypothalamus involving proopiomelanocortin (POMC) neurons [87]. Obese people secrete less PYY than non-obese people and have relatively lower levels of serum ghrelin [88]. Thus, PYY replacement may be used to treat overweight and obesity [88,89]. Indeed, caloric intake during a buffet lunch offered two hours after PYY infusion was decreased by 30% in obese subjects (*p* < 0.001) and 31% in lean subjects (*p* < 0.001) [89]. The extent of reduction was quite impressive in the former case. Although obese persons are shown to have lower circulating levels of PYY postprandially, they also seem to display normal sensitivity to the anorectic effect of PYY3-36. Taken together, obesity may bias the PYY sensitivity issue, and the anorectic effect of PYY could serve as a therapeutic mechanism for developing anti-obesity drugs [90].

### 4.5. Glucagon-Like Peptide 1 (GLP-1)

GLP-1 is a key hormone co-released with PYY from the distal intestinal L-cells of the gut after a meal. It is secreted in two equally potent forms, GLP-1 (7–37) and GLP-1 (7–36) [91]. GLP-1 primarily functions to stimulate glucose-dependent insulin secretion, enhance β-cell growth and survival, inhibit glucagon release, and suppress food intake [92]. Peripheral administration of GLP-1 decreases food intake and increases fullness in humans in part by slowing gastric emptying and promoting gastric distension [93]. Plasma levels of GLP-1 are higher before and after food intake in lean as compared to obese individuals, while the latter are associated with lower fasting GLP-1 and an attenuated postprandial release [94]. Restrictive bariatric procedures are an effective means of reducing obesity. Currently, data are limited regarding changes in GLP-1 concentrations in obese patients after surgeries [95].

### 4.6. Cholecystokinin (CCK)

Cholecystokinin (CCK), an endogenous peptide hormone present in the gut and the brain, helps control appetite, ingestive behavior, and gastric emptying via both peripheral and central mechanisms. CCK also impacts physiological processes related to anxiety, sexual behavior, sleep, memory, and intestinal inflammation [95]. CCK represents a collection of hormones varied by the arbitrary numbering of particular amino acids (for example, CCK 8 in the brain, and CCK 33 and CCK 36 in the gut). These various hormones do not appear to differ significantly in physiological functions. CCK originating from the gut is rapidly released from the duodenal and jejunal mucosa in response to nutrients’ ingestion peaks at about 15–30 min postprandially, and remains elevated for up to 5 h [96]. It is a potent stimulator of pancreatic digestive enzymes and bile from the gallbladder [63]. CCK delays gastric emptying and promotes intestinal motility. As a neuropeptide, CCK activates receptors on vagal afferent neurons, which transmit satiety signals to the dorsomedial hypothalamus. This action suppresses orexigenic neuropeptide NPY and provides feedback to reduce meal size and meal duration [97].

In summary, peripheral hormonal signals released from the GI tract (ghrelin, PYY, GLP-1, and CCK), pancreas (insulin), and adipose tissue (leptin) constitute a key component in the gut-brain axis-mediated control of appetite, energy expenditure, and obesity. While leptin and insulin may be considered more long-term regulators of energy balance, ghrelin, CCK, peptide YY, and GLP-1 are sensors related to meal initiation and termination and hence affect appetite and body weight more acutely. These hormones and peptides alter appetite and eating behaviors by acting on hypothalamic and brainstem nuclei and perhaps on the dopaminergic pathway in the midbrain reward center; they have demonstrated potential as therapeutic targets for anti-obesity treatments.

## 5. Neuroimaging Studies

Neuroimaging is a common tool to investigate the neurological basis of appetite and body weight regulation in humans in terms of cue-induced brain responses and structural analyses [98]. Neuroimaging studies are often used to examine alterations in brain responses to food intake and/or food cues, dopamine function, and brain anatomy in obese relative to lean individuals. Hyper- or hypo-activation in response to food intake or food cues in multiple brain regions implicated in reward (e.g., striatum, OFC, and insula), emotion and memory (e.g., AMY and hippocampus (HIPP)), homeostatic regulation of food intake (e.g., HPAL), sensory and motor processing (e.g., insula and precentral gyrus), and cognitive control and attention (e.g., prefrontal and cingulate cortex) have been found in obese *versus* normal weight subjects [98].

### 5.1. Functional Neuroimaging

By measuring brain responses to pictures of high-calorie foods (e.g., hamburgers), low-calorie foods (e.g., vegetables), eating-related utensils (e.g., spoons), and neutral images (e.g., waterfalls and fields), task fMRI studies have uncovered greater brain activation to high-calorie foods *versus* neutral images in the caudate/putamen (reward/motivation), anterior insula (taste, interception, and emotion), HIPP (memory), and parietal cortex (spatial attention) in obese female subjects relative to thin ones [99]. Moreover, the NAc, medial and lateral OFC, AMY (emotion), HIPP and MPFC (motivation and executive function), and ACC (conflict monitoring/error detection, cognitive inhibition, and reward-based learning) also exhibit enhanced activation in response to pictures of high-calorie foods *versus* non-foods and/or low-calorie food pictures [100]. These results illuminate the relationship between cortical responses to food cues and obesity and provide important insights into the development and maintenance of obesity [101].

Dysfunctional food cue-related brain activity involves not only the reward/motivation areas, but also neural circuits implicated in inhibitory control and in the limbic area. A PET study noted attenuated decreases in hypothalamic, thalamic, and limbic/paralimbic activity in obese (BMI ≥ 35) relative to lean (BMI ≤ 25) males [101]. Soto-Montenegro *et al*. and Melega *et al.* [102,103] investigated changes in brain glucose metabolism after deep brain stimulation (DBS) in the lateral hypothalamic area (LHA) in a rat model of obesity using PET-CT imaging. They found that average food consumption during the first 15 days was lower in DBS-treated animals than in non-stimulated animals. DBS increased metabolism in the mammillary body, subiculum hippocampal area, and AMY, while a decrease in metabolism was recorded in the thalamus, caudate, temporal cortex, and cerebellum [102,104]. DBS produced significant changes in brain regions associated with the control of food intake and brain reward, presumably by ameliorating the impaired hippocampal functioning seen in obese rats. The smaller weight gain in the DBS group suggests that this technique could be considered as an option for the treatment of obesity [102]. Both PET and SPECT have been used to study brain abnormality under various conditions [105,106,107,108,109,110,111].

Greater activation in the ventromedial, dorsomedial, anterolateral, and dorsolateral PFC (dlPFC; cognitive control) regions was reported after a nutritionally complete (50% of daily Resting Energy Expenditure (REE) provided) liquid meal administration following a 36 h fast in a PET study [101], although further analysis and collection of additional data using a different meal paradigm disputed these findings. On the other hand, lessened postprandial activation in the dlPFC in obese (BMI ≥ 35) *versus* lean (BMI ≤ 25) adults was consistently observed in this and other studies [112]. A study of older adults discovered a significant correlation between higher levels of abdominal fat/BMI and reduced fMRI activation to sucrose in DA-related brain regions, and between hypo-reward response and obesity in older adults as opposed to young adults [98]. Taken together, decreased dopamine function offers one plausible explanation for weight and fat gain in older adults [113]. The general implication from these studies is that obesity is consistently linked to abnormal responses to visual food cues in a disturbed network of brain regions indicated in reward/motivation and emotion/memory control. Overeating in obese individuals may be related to a combination of sluggish homeostatic responses to satiety in the hypothalamus, and a reduction in DA pathway activities and inhibitory response in the dlPFC [98].

Despite the progress in our understanding of neuro-circuitry control of overeating and obesity, it remains unknown whether the deficits in the control mechanisms actually precede or follow overeating or obesity. Longitudinal neuroimaging studies in rodent models of acquired diet-induced obesity (*i.e.*, comparing imaging results before, during, and after the development of dietary obesity and/or following caloric restriction after obesity establishment) and in obese humans before and after bariatric surgery, which successfully curtails overeating and reduces obesity, may provide important insights into a causal or consequential relationship between overeating (or obesity) and dysfunctional neural circuit regulation.

### 5.2. Structural Imaging

Recent evidence indicates brain anatomical structural changes related to obesity development [114]. For instance, morphometric analysis of MRI uncovered an association between greater body weight and lower total brain volume in humans [115]. In particular, high BMI results in decreased gray matter (GM) volumes in the frontal cortex, including OFC, right inferior, and middle frontal cortex, and is negatively correlated with frontal GM volumes [116,117,118] and a larger right posterior region encompassing the parahippocampal (PHIPP), fusiform, and lingual gyri [114]. One study with 1428 adults also observed a negative correlation, in males, between BMI and overall GM volume, as well as in bilateral medial temporal lobes, occipital lobes, precuneus, putamen, postcentral gyrus, midbrain, and anterior lobe of the cerebellum [116,118]. A separate study of cognitively normal elderly subjects who were obese (77 ± 3 years), overweight (77 ± 3 years), or lean (76 ± 4 years) reported reduced volume in the thalamus (sensory relay and motor regulation), HIPP, ACC, and frontal cortex [119]. These reported brain structural changes were based on cross-sectional data in adults, but it remains unclear whether the changes precede or follow obesity. Nonetheless, the volume reductions in areas associated with reward and control might be consequential to impaired functional activation in relation to obesity and may help explain the phenotypic overeating in obesity. Reduced volume in structures such as the HIPP may in part underlie the higher rates of dementia [120,121] and cognitive decline [122] in obese individuals. Sleep apnea [123], increased secretion of adipocyte hormones such as leptin [124], or release of pro-inflammatory factors due to high-fat consumption may be physiological factors mediating the changes in the brain [125]. These findings imply that hedonic memories of eating certain foods may be critically important in the regulation of feeding [98,126]. Purnell *et al*. [127] found that hyperphagia and obesity may be related to damages to the hypothalamus in humans. Indeed, a female patient in this study with a brainstem cavernoma that damaged structural pathways experienced a sudden onset of hyperphagia and weight gain of more than 50 kg in the space of less than a year following surgical drainage via a midline suboccipital craniotomy. Diffusion tensor imaging revealed loss of nerve fiber connections between her brainstem, hypothalamus, and higher brain centers but preservation of motor tracks. Karlsson *et al*. [128] studied 23 morbidly obese subjects and 22 non-obese volunteers by using voxel-based analysis of diffusion tensor imaging and of T1-weighted MRI images. Full-volume statistical parametric mapping analysis was used to compare fractional anisotropy (FA) and mean diffusivity (MD) values as well as gray (GM) and white matter (WM) density between these groups [128]. Results indicated that obese subjects had lower FA and MD values and lower focal and global GM and WM volumes than control subjects. The focal structural changes were observed in brain regions governing reward seeking, inhibitory control, and appetite. Regression analysis showed that FA and MD values as well as GM and WM density were negatively associated with body fat percentage. Moreover, the volume of abdominal subcutaneous fat was negatively associated with GM density in most regions [128].

## 6. Brain Circuits Related to Obesity

**Figure 1:**
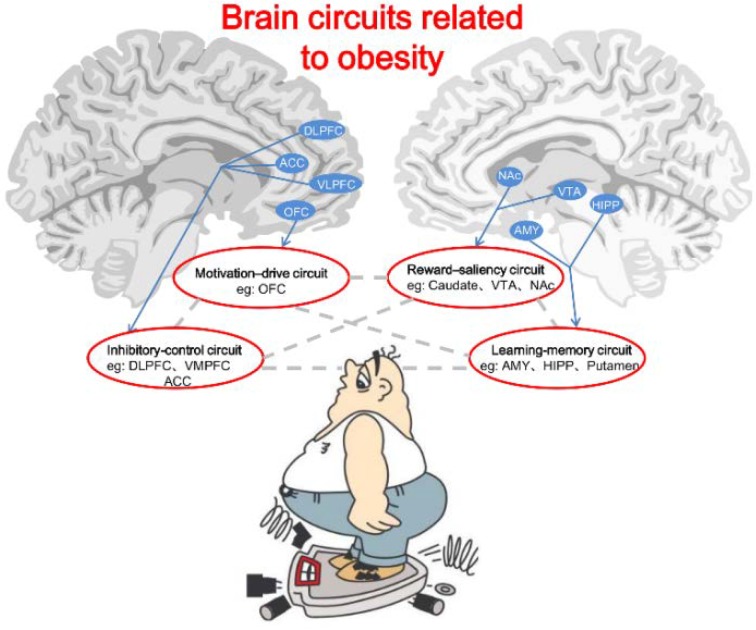
Brain circuits related to obesity. The circuits include motivation-drive (e.g., OFC), reward-saliency (e.g., VTA and NAc), inhibitory-control (e.g., DLPFC, ACC, and VMPFC) and learning-memory (e.g., AMY, HIPP, and Putamen). Gray dotted lines represent functional interactions between the brain circuits. In this model, during exposure to the reinforcer (*i.e.*, foods) or to the cues conditioned to the reinforcer, there appears to be an obesity-related lower perception of reward (processed by the learning-memory circuit), which promotes overactivation of the reward-saliency and motivation-drive circuits while decreasing the inhibitory-control circuit activity. The overall outcome in obese persons is a lessened ability or an inability to inhibit the drive to seek and consume foods.

Abbreviations: OFC: orbital frontal cortex; VTA: ventral tegmental area; NAc: nucleus accumbens; DLPFC: dorsal lateral prefrontal cortex; ACC: anterior cingulate gyrus; VMPFC: ventromedial prefrontal cortex; AMY: amygdala; HIPP: hippocampus.

Brain imaging studies have provided ample evidence for an imbalance between neural circuits that motivate behaviors (because of their involvement in reward and conditioning) and the circuits that control and inhibit prepotent responses in overeating cases. A neurocircuitry-based model for obesity has formed based on the study results [129]. The model involves four main identified circuits: (i) reward-saliency; (ii) motivation-drive; (iii) learning-memory; and (iv) inhibitory-control circuit [130] (Figure 1). In vulnerable individuals, consumption of palatable foods in large quantities may disturb the normal balanced interaction among these circuits, resulting in an enhanced reinforcing value of foods and a weakening of inhibitory control. Prolonged exposure to high-calorie diets may also directly alter conditioned learning and therefore reset reward thresholds in at-risk individuals. The ultimate changes in cortical top-down networks that regulate prepotent responses lead to impulsivity and compulsive food intake.

### 6.1. Reward-Saliency Circuit

Many obese individuals demonstrate hyporesponsivity of the reward circuitry, which induces compensatory overeating to achieve sufficient reward [58,63]. Consumption of palatable foods activates many brain regions that respond to food receipt and encode the relative perceived pleasantness of foods, such as the midbrain, insula, dorsal striatum, subcallosal cingulate, and PFC. Chronic exposure to palatable foods diminishes satiety and food pleasantness [92,131]. Dopamine is a neurotransmitter critical for reward processing, motivation, and positive behavior reinforcement [31,61], and plays an important role in the reward-saliency circuit. The mesolimbic DA projection from the ventral tegmental area (VTA) to the NAc encodes reinforcement for feeding [132,133]. DA release in the dorsal striatum can directly impact food ingestion, and the magnitude of the release correlates with ratings of meal pleasantness [99]. Volkow *et al*. [129] adopted PET and a multiple tracer approach to examine the DA system in healthy controls, in subjects with drug addiction, and in morbidly obese individuals, showing that both addiction and obesity are associated with decreased DA dopamine 2 (D2) receptor availability in the striatum. The tendency to eat during periods of negative emotions was negatively correlated with D2 receptor availability in the striatum in normal weight subjects—the lower the D2 receptors, the higher the likelihood that the subject would eat if emotionally stressed [134]. In another study, DA agonist administration increased the portion size of meals and length of feeding, while long-term DA supplements boosted body mass and feeding behavior [135]. Morbidly obese subjects have shown a higher level of baseline metabolism than usual in the somatosensory cortex [136]. This is a brain area that directly influences DA activity [137,138,139]. D2 receptors have important functions in reward seeking, prediction, expectation, and motivation-related feeding and addictive behaviors [140]. D2 receptor antagonists block food-seeking behaviors that are dependent upon either the palatable foods themselves or the reinforcement of the cues-induced anticipation of the rewards [141]. According to Stice *et al*. [35] individuals may overeat to compensate for a hypofunctioning dorsal striatum, particularly those with genetic polymorphisms (TaqIA A1 allele) thought to attenuate dopamine signaling in this region. Along the same line, the tendency to overeat in the normal weight individuals with negative emotions was found to be negatively correlated with D2 receptor levels [134]. Wang [142] and Haltia [143] discovered that the lower D2 receptors correlated with higher BMI in morbidly obese (BMI > 40) and obese subjects, respectively. These findings are consistent with the notion that diminished D2 receptor activity promotes feeding and the risk for obesity [144]. Guo *et al*. [145] found that obesity and opportunistic eating were positively associated with D2-like receptor binding potential (D2BP) in the dorsal and lateral striatum, the sub-regions supporting habit formation. Conversely, a negative relation between obesity and D2BP was observed in the ventromedial striatum, a region supporting reward and motivation [145].

### 6.2. Motivation-Drive Circuit

Several areas of the prefrontal cortex, including the OFC and CG, have been implicated in motivation of food consumption [146]. Abnormalities in these regions may enhance eating behaviors that are dependent on sensitivity to the reward and/or established habits of the subject. Obese people display increased activation of prefrontal regions upon exposure to a meal [101]. Moreover, they also respond to food-cues with activation of the medial prefrontal cortex and cravings [49]. Sucrose also excites the OFC, a region responsible for “scoring” the reward value of a food or any other stimulus, more so in the obese patients as compared to lean controls. The structural abnormality of the OFC, presumably affecting the reward processing and self-regulatory mechanisms, may play a crucial role in binge eating disorder and bulimia nervosa [147]. Not surprisingly, the aberrant eating behaviors may share common neural circuitry regulation with drug addiction. For example, Volkow *et al*. [148] propose that exposure to drugs or drug-related stimuli in the withdrawal state reactivates the OFC and results in compulsive drug intake. A similar result about the OFC was noted in a separate study. Further evidence highlights the OFC influence on compulsive disorders [149]. For instance, damage of the OFC leads to a behavioral compulsion to procure the reward even when it is no longer reinforcing [149]. This is consistent with the accounts of drug addicts who claim that once they start taking the drug they cannot stop, even when the drug is no longer pleasurable [98].

### 6.3. Learning-Memory Circuit

A place, a person, or a cue can trigger memories of a drug or food and powerfully affect addictive behaviors, which underscores the importance of learning and memory in addiction. Memories can produce an intense desire for the drug or food (a craving) and frequently result in relapse. Multiple memory systems have been proposed in drug or food addiction, including conditioned incentive learning (mediated in part by the NAc and the AMY), habit learning (mediated in part by the caudate and the putamen), and declarative memory (mediated in part by the HIPP) [150]. Conditioned incentive learning about neutral stimuli or exaggerated stimulation by overeating generates reinforcing properties and motivational salience even in the absence of food. Through habit learning, well-learned sequences of behaviors are elicited automatically in response to appropriate stimuli. Declarative memory is more about the learning of affective states in relationship to food intake [149]. Multiple PET, fMRI, and MRI studies have investigated brain responses to food intake and food cues with respect to dopamine function and brain volume in lean *versus* obese individuals and identified irregularities in emotion and memory circuits (e.g., AMY and HIPP) [98]. For example, some satiety signaling generated from homeostatic areas is impaired (e.g., delayed fMRI inhibition response in the hypothalamus) while hunger signals from emotion/memory areas and sensory/motor areas (e.g., greater activation in AMY, HIPP, insula, and precentral gyrus in response to food cues) are heightened in obese individuals [98]. Hippocampal function has been implicated in memories of foods or the rewarding consequences of eating in humans and rodents. If this function is disturbed, retrieval of memories and environmental cues may evoke more powerful appetitive responses essential to obtaining and consuming foods [151]. In drug-related addiction, memory circuits set the expectations of the drug’s effects and thus affect the effectiveness of drug intoxication. Activation of brain regions linked to memory has been indicated during drug intoxication [152,153] and craving induced by drug exposure, video, or recall [154,155,156]. Habit learning involves the dorsal striatum and DA release in this area [157]. Drug abusers have decreased D2 receptor expression and decreased DA release in the dorsal striatum during withdrawal [149]. In animals, prolonged drug exposure induces changes in the dorsal striatum more persistent than those in the NAc, which has been interpreted as a further progression into the addicted state [158].

### 6.4. Inhibitory-Control Circuit

The brain top-down control system constitutes a network of frontal brain regions involved in executive control, goal-directed behavior, and response inhibition [159]. The dlPFC and inferior frontal gyrus (IFG) are components of the system that are significantly activated during an individual’s conscious effort to adjust their desire to consume subjectively palatable but realistically unhealthy foods [160]. Such dlPFC and IFG activities function to inhibit the desire to consume food, as evidenced by greater cortical activation in those areas that correlate with better self-control in choosing between healthy and unhealthy foods [161]. Obese individuals with PWS, a genetic disorder characterized by profound hyperphagia, demonstrate reduced activity in the dlPFC post-meal as compared to non-diseased obese individuals [162]. Collectively, inhibitory control of food consumption seems to rely on the ability of the brain’s top-down control systems to modulate the subjective valuation of food. Individual differences in food intake regulation may result from structural differences of the dlPFC and/or connectivity with brain valuation regions [161]. Indeed, while obese subjects showed reduced inhibitory response in the dlPFC [98], drug-addicted individuals also displayed abnormalities in the PFC, including the anterior CG [163]. The PFC plays a role in decision making and in inhibitory control [164]. Disruption of the PFC may lead to inadequate decisions that favor immediate rewards over delayed but more satisfying responses. It could also contribute to impaired control over drug intake in spite of the addict’s desire to refrain from taking the drug [163]. Thus, deficiencies in self-monitoring and decision-making processes in drug addiction [165,166] are presumably associated with disrupted prefrontal functions. In support of this notion, preclinical studies unveiled a significant increase in dendritic branching and the density of dendritic spines in the PFC following chronic administration of cocaine or amphetamine [167]. The changes in synaptic connectivity could result in poor decision making, judgment, and cognitive control in drug addiction. This kind of alteration in prefrontal activation has in fact been observed during a working memory task in smokers compared with ex-smokers [168]. In this regard, Goldstein *et al*. [163] previously proposed that disruption of the PFC could cause loss of self-directed/willed behavior in favor of automatic sensory-driven behavior. More specifically, drug intoxication likely exacerbates troubled behaviors due to loss of the inhibitory control that the prefrontal cortex exerts over the AMY [169]. Disinhibition of the top-down control frees behaviors normally kept under close monitoring and simulates stress-like reactions in which control is lifted and stimulus-driven behavior is facilitated [163].

## 7. Therapeutic Interventions

A number of medical and surgical strategies are available to treat obesity besides the typical combination of diet, exercise, and other behavioral modifications. Weight loss drugs may take effect by preventing fat absorption or suppressing appetite. Certain surgical weight loss procedures such as the Roux-en-Y gastric bypass (RYGB) alter brain-gut interaction and mediate weight loss. Fecal microbiota transplantation (FMT), infusion of a fecal suspension from a healthy individual into the gastrointestinal (GI) tract of another person, has been used successfully not only for alleviating recurrent *Clostridium difficile* infection, but also for GI and non-GI-related diseases such as obesity.

### 7.1. Dietary and Lifestyle Interventions

Dietary and lifestyle interventions aimed at decreasing energy intake and increasing energy expenditure through a balanced dietary and exercise program are an essential component of all weight management programs [170]. Diets are based on the principles of metabolism and work by reducing the intake of calories (energy) to create a negative energy balance (*i.e.*, more energy is used than is consumed). Diet programs may produce weight loss over the short term [171,172], but maintaining this weight loss is frequently difficult and often requires making exercise and a lower-energy diet a permanent part of a person’s lifestyle [173]. Physical exercise is an integral part of a weight management program, especially for weight maintenance. With use, muscles consume energy derived from both fat and glycogen. Due to the large size of leg muscles, walking, running, and cycling are the most effective means of exercise for reducing body fat [174]. Exercise affects macronutrient balance. During moderate exercise, equivalent to a brisk walk, there is a shift to greater use of fat as a fuel [175,176]. The American Heart Association recommends a minimum of 30 min of moderate exercise at least five days a week in order to maintain health [177]. As with dietary treatment, many physicians do not have the time or expertise to advise patients on an exercise program that is tailored to individual needs and capabilities. The Cochrane Collaboration found that exercise alone led to limited weight loss. In combination with diet, however, it resulted in a 1 kilogram weight loss over dieting alone. A 1.5 kilogram (3.3 lb) loss was observed with a greater degree of exercise [178,179]. Success rates of long-term weight loss maintenance with lifestyle changes are low, ranging from 2% to 20% [180]. Dietary and lifestyle changes are effective in limiting excessive weight gain in pregnancy and improve outcomes for both the mother and the child [181]. Lifestyle interventions remain the cornerstone of obesity treatment, but adherence is poor and long-term successes modest because of significant barriers both on the part of affected individuals and health care professionals responsible for the treatment.

### 7.2. Weight Loss Drugs

To date, four weight loss drugs have been approved by the US Food and Drug Association (FDA): Xenical, Contrave, Qsymia, and Lorcaserin [4]. These medicines are divided into two types. Xenical is the only fat absorption inhibitor. Xenical acts as a lipase inhibitor, which decreases the absorption of fats from the human diet by 30%. It is intended for use in conjunction with a healthcare provider-supervised regimen of caloric restriction [182].

Another type, which includes the other three medications, acts on the CNS as an “appetite suppressant.” The newly approved (in 2012) drug Lorcaserin, for example, is a selective small molecule agonist of the 5HT2C receptor. It was developed based on the anorexigenic property of the receptor to mediate weight loss [183]. Activation of 5HT2C receptors in the hypothalamus stimulates pro-opiomelanocortin (POMC) production and promotes satiety. A 5-HT2C receptor agonist regulates appetite behavior through the serotonin system [54]. Use of Lorcaserin is associated with significant weight loss and improved glycemic control in patients with type 2 diabetes mellitus [183]. The other two medications, Contrave and Quexa, target the DA reward system. Contrave is a combination of two approved drugs—bupropion and naltrexone. Either drug alone produces modest weight loss, while the combination exerts a synergistic effect [184]. Qsymia (Quexa) consists of two prescription drugs, phentermine and topiramate. Phentermine has been used effectively for years to reduce obesity. Topiramate has been used as an anti-convulsant in epilepsy patients, but induced weight loss in people as an accidental side effect [54]. Qsymia suppresses appetite by making people feel full. This property is particularly helpful for obese patients because it deters overeating and encourages compliance with a sensible eating plan.

### 7.3. Bariatric Surgery

Some obese patients may benefit from the weight loss drugs with a limited efficacy, but they are often afflicted by side effects. Bariatric surgery (adjustable gastric banding (AGB), Roux-en Y gastric bypass (RYGB), or laparoscopic sleeve gastrectomy (LSG)) [185] represents the only current form of treatment for overt obesity with established long-term effectiveness [186]. Bariatric surgery alters the gut hormone profile and neural activity. Understanding the mechanisms underlying neurophysiological and neuroendocrine changes with the surgery will advance the development of non-surgical interventions to treat obesity and related comorbidities, which could be a viable alternative for obese individuals who do not have access or do not qualify for the surgery. RYGB is the most frequently performed bariatric procedure, providing significant and sustained weight loss at long-term follow-up [187]. However, the mechanisms of action in RYGB that result in weight loss are not well understood. A significant proportion of the resulting reduction in caloric intake is unaccounted for by the restrictive and malabsorptive mechanisms and is thought to be mediated by neuroendocrine function [188]. RYGB is thought to cause substantial and simultaneous changes in gut peptides [95,189], brain activation [95,190], the desire to eat [190], and taste preferences. For example, postsurgical reductions in ghrelin and earlier and enhanced postprandial elevations of PYY and GLP-1 may reduce hunger and promote satiety [191]. Relative to changes in gut peptides, very little is known about changes in brain activation following bariatric procedures. Investigations of non-surgical weight loss support an increase in reward-related/hedonic activation in response to appetitive cues [95], which helps explain weight regain in dieters. In contrast, the absence of an increase in desire to eat following RYGB, even on exposure to highly palatable food cues, is striking, and consistent with systemic changes in neural responses to food cues. Ochner *et al*. [188] used fMRI and verbal rating scales to assess brain activation and desire to eat in response to high- and low-calorie food cues in 10 female patients, one month before and post-RYGB surgery. The results demonstrated postsurgical reductions in brain activation in key areas within the mesolimbic reward pathway [188]. There was also a greater surgical-induced reduction in conjoint (visual + auditory) whole-brain activation in response to high-caloric foods than in response to low-caloric foods, especially in corticolimbic areas within the mesolimbic pathway including the VTA, ventral striatum, putamen, posterior cingulate, and dorsal medial prefrontal cortex (dmPFC) [188]. This is in contrast to heightened food responses to high caloric contents in regions such as the cingulate gyrus, thalamus, lentiform nucleus and caudate, ACC, medial frontal gyrus, superior frontal gyrus, inferior frontal gyrus, and middle frontal gyrus before the surgery [188]. These changes mirrored concurrent postsurgical reductions in the desire to eat, which were greater in response to food cues that were high in caloric density (*p* = 0.007). These RYGB surgery-related occurrences provide a potential mechanism for the selective reduction in preferences for high-calorie foods, and suggest partial neural mediation of changes in caloric intake following surgery [185,188]. These changes may be in part directly related to an altered perception of reward [192]. Halmi *et al*. [193] noted a statistically significant decrease in intake of high-fat meats and high-calorie carbohydrates six months after gastric bypass. Patients found these foods were no longer enjoyable. Some bypass patients even avoided high-fat food [194], while others lost interest in sweets or desserts after surgery [195,196,197,198]. Decrease in taste thresholds for foods, such as blunted recognition of sweetness or bitterness, has been reported after bariatric surgery [192,199]. Moreover, altered brain dopamine signaling was discovered after bariatric surgery. Whereas D2 receptors were reduced in the caudate, putamen, ventral thalamus, HPAL, substantianigra, medial HPAL, and AMY after RYGB and sleeve gastrectomy, an increase in D2 receptors was found in the ventral striatum, caudate, and putamen that was proportional to the weight lost [131,200,201]. The discrepancy in results may be due to the presence of comorbid conditions which can alter dopamine signaling [192]. Overall, bariatric surgery, especially the RYGB procedure, is currently the most effective long-term treatment for obesity and its associated comorbidities. More investigations are warranted to examine how the gut*-*brain axis mediates the remarkable surgical effects on the control of reward-based eating behavior [202].

### 7.4. Fecal Microbiota Transplantation

Mounting evidence pinpoints an apparent function of the gut microbiota in the regulation of energy balance and weight maintenance in animals and humans. Such a function influences the development and progression of obesity and other metabolic disorders including type 2 diabetes. Manipulation of the gut microbiome represents a novel approach to the treatment of obesity over and above the diet and exercise strategies [203]. A new form of intervention, fecal microbiota transplantation (FMT), was recently introduced into clinical treatment for obesity [204]. The intestinal microbiotas metabolize ingested nutrients into energy-rich substrates for utilization by the host and commensal flora [203,204] and adapt metabolically based on nutrient availability. After comparing the distal gut microbiota profiles of genetically obese mice and their lean littermates, and that of obese people and lean volunteers, it was found that obesity varies with the relative abundance of the two dominant bacterial divisions, the Bacteroidetes and the Firmicutes. Both metagenomic and biochemical analyses provide an understanding of the influence of these bacteria on the metabolic potential of the mouse gut microbiota. Specifically, the obese microbiome has an increased capacity to harvest energy from the diet. Furthermore, the trait is transmissible: colonization of germ-free mice with an “obese microbiota” results in a significantly enlarged total body fat mass than colonization with a “lean microbiota”. These findings identify the gut microbiota as an important contributing factor to the pathophysiology of obesity [203,205]. Indeed, different studies reported a 60% increase in body fat, insulin resistance, and the overall obese phenotype transmission following introduction of intestinal microbiota from conventionally raised mice to germ-free mice [206]. Data in this regard are sparse thus far in humans. One double-blind, controlled trial randomized 18 men with metabolic syndrome to undergo FMT. They were given either their own feces or feces donated from lean males [207]. The nine men who received stool from lean donors developed markedly reduced fasting triglyceride levels and enhanced peripheral insulin sensitivity compared with those who were transplanted with their own (placebo) stool [207].

## 8. Conclusions

Much progress has been made in recent years toward an understanding of obesity from the perspectives of epidemiology, food addiction, neurohormonal and endocrine regulation, neuroimaging, pathological neurochemical control, and therapeutic interventions. Overconsumption of calorie-dense foods is one significant causal factor in obesity, which may provoke the food addiction mechanism. Obesity may result from a combination of dysfunction of brain circuits and neuroendocrine hormones related to pathological overeating, physical inactivity and other pathophysiological conditions. New therapeutic strategies have become available for managing obesity apart from the standard protocol of diet and/or exercise. These include anti-obesity drugs, various bariatric surgical procedures, and FMT. Despite significant progress, obesity remains a pressing public health challenge and warrants urgent and unwavering research efforts to illuminate the neuropathophysiological basis of the chronic disease.
